# α-Synuclein expression in response to bacterial ligands and metabolites in gut enteroendocrine cells: an *in vitro* proof of concept study

**DOI:** 10.1093/braincomms/fcad285

**Published:** 2023-10-24

**Authors:** Michael J Hurley, Elisa Menozzi, Sofia Koletsi, Rachel Bates, Matthew E Gegg, Kai-Yin Chau, Hervé M Blottière, Jane Macnaughtan, Anthony H V Schapira

**Affiliations:** Department of Clinical and Movement Neurosciences, UCL Queen Square Institute of Neurology, London NW3 2PF, UK; Aligning Science Across Parkinson’s (ASAP) Collaborative Research Network, Chevy Chase, MD 20815, USA; Department of Clinical and Movement Neurosciences, UCL Queen Square Institute of Neurology, London NW3 2PF, UK; Aligning Science Across Parkinson’s (ASAP) Collaborative Research Network, Chevy Chase, MD 20815, USA; Department of Clinical and Movement Neurosciences, UCL Queen Square Institute of Neurology, London NW3 2PF, UK; Aligning Science Across Parkinson’s (ASAP) Collaborative Research Network, Chevy Chase, MD 20815, USA; Department of Clinical and Movement Neurosciences, UCL Queen Square Institute of Neurology, London NW3 2PF, UK; Aligning Science Across Parkinson’s (ASAP) Collaborative Research Network, Chevy Chase, MD 20815, USA; Department of Clinical and Movement Neurosciences, UCL Queen Square Institute of Neurology, London NW3 2PF, UK; Aligning Science Across Parkinson’s (ASAP) Collaborative Research Network, Chevy Chase, MD 20815, USA; Department of Clinical and Movement Neurosciences, UCL Queen Square Institute of Neurology, London NW3 2PF, UK; Aligning Science Across Parkinson’s (ASAP) Collaborative Research Network, Chevy Chase, MD 20815, USA; Aligning Science Across Parkinson’s (ASAP) Collaborative Research Network, Chevy Chase, MD 20815, USA; Université Paris-Saclay, INRAE, MetaGenoPolis, Jouy en Josas, & Nantes Université, INRAE, UMR 1280 PhAN, Nantes 44093, France; Department of Clinical and Movement Neurosciences, UCL Queen Square Institute of Neurology, London NW3 2PF, UK; Aligning Science Across Parkinson’s (ASAP) Collaborative Research Network, Chevy Chase, MD 20815, USA; Institute for Liver and Digestive Health, University College London, London NW3 2PF, UK; Department of Clinical and Movement Neurosciences, UCL Queen Square Institute of Neurology, London NW3 2PF, UK; Aligning Science Across Parkinson’s (ASAP) Collaborative Research Network, Chevy Chase, MD 20815, USA

**Keywords:** toll-like receptors, free fatty acid receptors, short-chain fatty acids, enteroendocrine cells, microbiome

## Abstract

Caudo-rostral migration of pathological forms of α-synuclein from the gut to the brain is proposed as an early feature in Parkinson’s disease pathogenesis, but the underlying mechanisms remain unknown. Intestinal epithelial enteroendocrine cells sense and respond to numerous luminal signals, including bacterial factors, and transmit this information to the brain via the enteric nervous system and vagus nerve. There is evidence that gut bacteria composition and their metabolites change in Parkinson’s disease patients, and these alterations can trigger α-synuclein pathology in animal models of the disorder. Here, we investigated the effect of toll-like receptor and free fatty acid receptor agonists on the intracellular level of α-synuclein and its release using mouse secretin tumour cell line 1 enteroendocrine cells. Secretin tumour cell line 1 enteroendocrine cells were treated for 24 or 48 h with toll-like receptor agonists (toll-like receptor 4 selective lipopolysaccharide; toll-like receptor 2 selective Pam3CysSerLys4) and the free fatty acid receptor 2/3 agonists butyrate, propionate and acetate. The effect of selective receptor antagonists on the agonists’ effects after 24 hours was also investigated. The level of α-synuclein protein was measured in cell lysates and cell culture media by western blot and enzyme-linked immunosorbent assay. The level of α-synuclein and tumour necrosis factor messenger RNA was measured by quantitative reverse transcription real-time polymerase chain reaction. Stimulation of secretin tumour cell line 1 enteroendocrine cells for 24 and 48 hours with toll-like receptor and free fatty acid receptor agonists significantly increased the amount of intracellular α-synuclein and the release of α-synuclein from the cells into the culture medium. Both effects were significantly reduced by antagonists selective for each receptor. Toll-like receptor and free fatty acid receptor agonists also significantly increased tumour necrosis factor transcription, and this was effectively inhibited by corresponding antagonists. Elevated intracellular α-synuclein increases the likelihood of aggregation and conversion to toxic forms. Factors derived from bacteria induce α-synuclein accumulation in secretin tumour cell line 1 enteroendocrine cells. Here, we provide support for a mechanism by which exposure of enteroendocrine cells to specific bacterial factors found in Parkinson’s disease gut dysbiosis might facilitate accumulation of α-synuclein pathology in the gut.

## Teaser

Hurley *et al*. report that exposure to bacterial membrane components (lipopolysaccharide and lipopeptides) and bacterial by-products (short-chain fatty acids) increases α-synuclein protein levels in mouse secretin tumour cell line 1 enteroendocrine cells. Enteroendocrine cells could therefore represent an initial peripheral site of Parkinson’s disease pathology under specific gut microbial conditions.

## Graphical Abstract

**Graphical Abstract:**
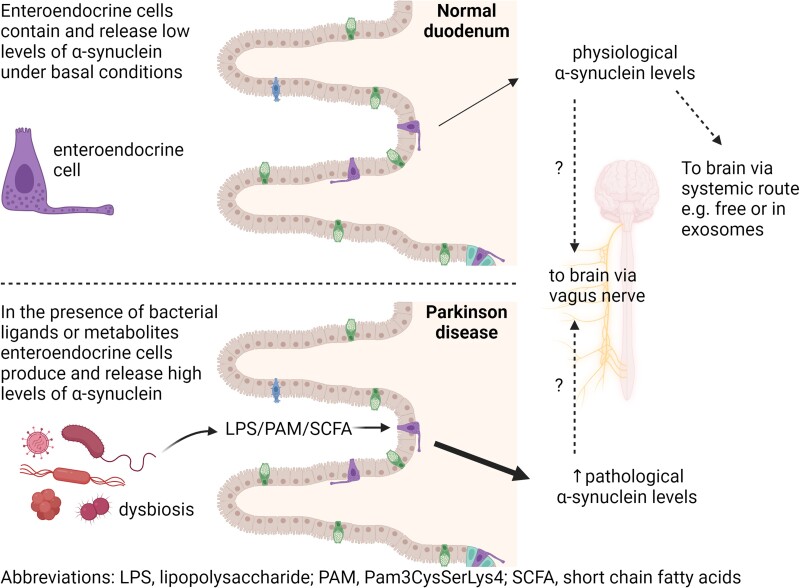

## Introduction

Parkinson’s disease is a progressive neurodegenerative disorder clinically characterized by bradykinesia, rigidity and resting tremor together with posture and gait abnormalities. Pathological features of Parkinson’s disease include degeneration of midbrain dopaminergic neurons in the substantia nigra pars compacta and loss of other catecholamine neurons in the brainstem and cholinergic neurons in the basal forebrain,^1,2^ together with increased levels^3^ and deposits of aggregated α-synuclein in the central nervous system and periphery.^4-6^

While motor symptoms are the cardinal features of Parkinson’s disease, non-motor symptoms adversely affect the quality of life for Parkinson’s disease patients. Among these, gastrointestinal disorders such as oropharyngeal/oesophageal dysphagia, gastroparesis and constipation frequently occur.^7-9^ The gastrointestinal tract may therefore play a central role in Parkinson’s disease pathogenesis, and it is now hypothesized that for some individuals a caudal to rostral spread of α-synuclein from the gastrointestinal tract to the brain could initiate the disease and underlie some of the manifestations affecting the gastrointestinal tract.^4,5^ Recently, it has been suggested that different subtypes of Parkinson’s disease, namely ‘gut-first’ and ‘brain-first’ subtypes, could exist. Within the former group, α-synuclein pathology might start in the periphery, within the enteric and parasympathetic nervous system, reflecting the presence of prodromal gastrointestinal disorders and subsequently spread to the sympathetic nervous system and lower brainstem.^10^ To demonstrate a ‘gut-first’ subtype, the trigger and location of the pathological cascade in the gastrointestinal tract in Parkinson’s disease need to be elucidated. Mounting evidence suggests that the trillions of bacteria living in the gut, the gut microbiome, could initiate Parkinson’s disease pathology. Experimentally, transfer of specific gut microbiome components and intestinal infection can cause Parkinson-like brain pathology in animal models^11-13^ and an altered gut microbiome composition was found in Parkinson’s disease patients.^14-18^ Intestinal infection could therefore act as a catalyst to promote a shift in the microbiome composition, allowing gastrointestinal microbial products and inflammatory mediators to increase the level of α-synuclein in the gut and subsequently the brain.^19^

A specific sub-population of intestinal epithelial cells—enteroendocrine cells—that are present in both the small intestine and colon are considered a potential candidate for initial gut pathology in Parkinson’s disease.^20^ Enteroendocrine cells comprise ∼1% of gut epithelium, express relatively high levels of α-synuclein,^21^ display neuronal-like properties including synaptic features^22^ and electrical excitability^23^ and are mono-synaptically connected with the brainstem through the vagus nerve.^24^ In contrast, α-synuclein is undetectable in enterocytes which make up the majority of the gut epithelium.^25^ Since enteroendocrine cells connect directly to enteric neurons and to the brainstem through the vagus nerve,^24^ they may act as a conduit for transfer of pathological forms of α-synuclein from the gut lumen to the brain via enteric innervation and the vagus nerve as depicted in the graphical abstract.^21,26^

Enteroendocrine cells express receptors for bacterial-derived components (toll-like receptors, TLR), and they respond to TLR agonists with increased proinflammatory cytokine production.^27-30^ Two studies have implicated a TLR-mediated response to bacterial ligands in Parkinson’s disease pathogenesis.^31^ TLR4 knockout mice exhibit attenuated motor dysfunction and neuro- and gastrointestinal inflammation and neurodegeneration in response to rotenone treatment.^32^ TLR2-deficient α-synuclein-overexpressing mice were found to have less neurodegeneration and neuronal α-synuclein concentrations.^33^

Enteroendocrine cells also express free fatty acid receptors (FFA) which recognize short-chain fatty acids (SCFA).^34,35^ SCFA (mainly acetate, propionate and butyrate) are key bacterial metabolites promoting host intestinal health, and they might modulate Parkinson’s disease pathogenesis.^12^ SCFA supplementation to germ-free α-synuclein-overexpressing mice induced changes in microglia and α-synuclein aggregation in the brain.^12^ However, sodium butyrate treatment ameliorated gut dysfunction and motor deficits in a rotenone-induced mouse model^36^ and increased α-synuclein mRNA in mouse secretin tumour cell line 1 (STC-1) enteroendocrine cells.^30^ Faecal levels of SCFA have also been found to be reduced in patients with Parkinson’s disease compared with age-matched controls.^37^ Here, we use mouse STC-1 enteroendocrine cells to assess the effect of treatment with TLR and FFA receptor agonists on α-synuclein expression and release.

## Materials and methods

### Mouse intestine

Ileal tissue from a 10-month *Gba* N370S colony wild-type mouse^38^ was removed and stored in 10% neutral buffered formalin (Fisher Scientific, 13191184) at 4°C for ≥4 days. Longitudinal pieces of gut tissue were embedded in paraffin wax (Thermo Fisher Scientific, 416770020) using a Shandon Excelsior^™^ tissue processor, mounted into plastic moulds (VWR, 720-0824) using a Leica wax embedding station and stored at room temperature. Sections (5 µm) were cut and dried onto Superfrost Plus^™^ adhesion microscope slides (Fisher Scientific, 10149870) and stored at room temperature. A detailed protocol is available via this link: dx.doi.org/10.17504/protocols.io.eq2ly72ywlx9/v1.

Animal husbandry and experimental procedures were performed in compliance with the UK Animal (Scientific Procedures) Act of 1986 at the University College London (UCL) biological resources facility.

### Cell culture

Mouse (*Mus musculus*) neuroendocrine duodenal adenoma STC-1 cells (RRID:CVCL_J405) were obtained from the American Type Culture Collection (ATCC-CRL-3254^™^) and grown in DMEM:F12 Ham with GlutaMax^™^ medium (Gibco^™^ 313310278) supplemented with 10% charcoal absorbed foetal bovine serum (LabTech, FB-1001F/500), 1 mM sodium pyruvate (Gibco^™^, 11360070), 100 μM non-essential amino acids (Gibco^™^, 11140050) and penicillin (100 U/ml) streptomycin (100 μg/ml) (Gibco^™^, 15070063) at 37°C under saturating humidity in a 5% CO_2_/95% air mixture (BOC, 225742-AV). A detailed protocol is available via this link: dx.doi.org/10.17504/protocols.io.5jyl8jky8g2w/v1.

For immunohistochemistry, cells were grown on 12-mm glass coverslips (Fisher Scientific, 11856933) treated with poly-D-lysine (Gibco^™^ A3890401) or Geltrex^™^ lactose dehydrogenase elevating virus (LDEV)-free reduced growth factor basement membrane matrix (Gibco^™^, A1413201). After treatments, cells were washed with phosphate buffered saline (PBS), fixed with 4% paraformaldehyde in PBS (Severn Biotech Ltd. 40-7401-05) for 15 min, washed twice with PBS and stored at −30°C. A detailed protocol is available via this link: dx.doi.org/10.17504/protocols.io.kqdg39yjpg25/v1.

Cell viability was assessed by resazurin reduction assay (RRA) using a CellTiter-Blue® Cell Viability Assay (Promega, G8080) according to the manufacturer’s instructions. Briefly, 20 µl/well of CellTiter-Blue® Reagent was added to each well of a 96-well plate containing treated STC-1 cells. The plate was incubated for 2 h, and then the fluorescence intensity was measured with Cytation 5 imaging multimode reader using a 560 excitation/590 emission filter set.

### Cell treatments

For TLR4 stimulation, cells were treated with 1 and 10 µg/ml of TLR4 selective ultrapure LPS-EB derived from *Escherichia coli* 0111:B4 (LPS) (InvivoGen, tlrl-3pelps) for 24 or 48 h. For TLR2 stimulation, cells were treated with 0.1 and 1 µg/ml of Pam3CSK4 (PAM) (Cambridge Bioscience, CAY24126) for 24 or 48 h. PAM is a selective ligand for the TLR2/TLR1 heterodimer.^39^ For antagonist experiments, cells were pre-treated for 1 h with 1 µM of the TLR4 inhibitor TAK-242 (Cambridge Bioscience, CAY13871)^40^ before addition of 10 µg/ml LPS for 24 h or 50 µM of the murine TLR2/1 signalling inhibitor C29 (Cambridge Bioscience, CAY27029)^41^ before addition of 1 µg/ml PAM for 24 h. Control groups were treated with agonists or antagonists alone for 24 h.

For FFA2/3 receptor stimulation, cells were treated with SCFA [4 mM sodium acetate (acetate)] (Merck, S8750), 4 mM sodium propionate (propionate) (Merck, P1880) and 2 mM sodium butyrate (butyrate) (Merck, B5887) for 24 h. The effect of 1 h pre-treatment with 1 µM GLPG0974 (Cambridge Bioscience, CAY28108) or 20 mM β-hydroxybutyrate (Cambridge Bioscience, CAY14148) on 24 h butyrate stimulation was tested. GLPG0974 is a potent antagonist of the human FFA2 receptor but has lower affinity for rodent FFA2 isoforms ([^3^H]GLPG0974, *K*_d_ 8.1 ± 0.9 and >150 nM, respectively).^42^ β-Hydroxybutyrate is an FFA3 receptor antagonist.^43^ Control groups were treated with antagonists or butyrate alone for 24 h. Following treatments, cell culture medium was removed and frozen and cells were washed with PBS and then frozen.

### Immunohistochemistry

Sections were processed for immunohistochemistry and staining visualized by immunofluorescence as previously described.^44^ Sections were heated at 60°C for 30 min, dewaxed (2 × 5 min) in Histo-Clear II (National Diagnostics), rehydrated through graded (100, 95, 80 and 70% *v*/*v*) ethanol solutions and washed in PBS for 5 min, then PBS containing 0.1% hydrogen peroxide for 5 min and then PBS again for 5 min. Antigen retrieval consisted of heating the sections in 15 mM sodium citrate buffer containing 0.1% Tween® 20, pH 6 for 25 min in an 800 watt microwave set on 30% power. After cooling with running tap water, sections were rinsed with PBS and then blocked for 90 min with 10% goat serum in PBS containing 0.005% Triton™ X-100 and 0.05% thimerosal (block buffer) followed by incubation with primary antibodies diluted in 10% goat serum containing 0.005% Triton^™^ X-100 and 0.05% thimerosal (antibody buffer) for 20 h. Sections were then washed with PBS (4 × 5 min) and then incubated fluorescently labelled secondary antibodies for 2 h. Sections were then washed again, incubated with 1 µg/ml 4′,6-diamidino-2-phenylindole (DAPI) for 5 min, washed again and then coverslipped with Hydromount^™^ (National Diagnostics). Details of antibodies are given in Table 1. A detailed protocol is available via this link: dx.doi.org/10.17504/protocols.io.eq2ly72ywlx9/v1.

**Table 1:** Primers and immunoglobulins

Primers (custom purchase from Integrated DNA Technologies: idtdna.com)				
*β-actin*				
Forward	5′-GCTGTCCCTGTATGCCTCTG-3′			
Reverse	5′-GATGTCACGCACGATTTCCC-3′			
*α-Synuclein*				
Forward	5′-CAGAGGCAGCTGGAAAGACA-3′			
Reverse	5′-CACCACTGCTCCTCCAACAT-3′			
*TNF*				
Forward	5′-GAGCCAGCGCGCCAACGCCCTCCT-3′			
Reverse	5′-TGAGGAGCACGTAGTCGGGGCAGC-3′			
**Reagent**	**Catalogue#**	**Supplier**	**IHH/western dilution**	**RRID** scicrunch.org/resources
*Primary antibodies*				
Rabbit anti-α-synuclein	ab212184	www.abcam.com/	1:500/1:1250	Not available
Rabbit anti-β-actin	ab241153	www.abcam.com/	–/1:5000	Not available
Rat anti-5-hydroxytryptamine	MAB352	www.merckmillipore.com/	1:200/–	RRID:AB_94865
Mouse anti-glucagon-like peptide-1	ab26278	www.abcam.com/	1:200/–	RRID:AB_470838
Mouse anti-cholecystokinin 8	ab37274	www.abcam.com/	1:200/–	RRID:AB_726010
*Secondary antibodies*				
Goat anti-rabbit IgG biotin	111-065-144-JIR	https://stratech.com	1:250/1:1000	RRID:AB_2337965
Goat anti-mouse IgG biotin	115-065-003-JIR	https://stratech.com	1:250/–	RRID:AB_2338557
Goat anti-rat IgG biotin	43R-1528-FIT	https://stratech.com	1:250/–	RRID:AB_10814469
Goat anti-mouse IgG Alexa Fluor™ Plus 555	A32727	www.thermofisher.com/	1:200/–	RRID:AB_2633276
Goat anti-rabbit IgG Alexa Fluor™ 488	A27034	www.thermofisher.com/	1:200/–	RRID:AB_2536097
Goat anti-rat IgG Alexa Fluor™ 546	A-11081	www.thermofisher.com/	1:200/–	RRID:AB_2534125
Streptavidin POD conjugate	11089153001	sigmaaldrich.com	1:250/1:1000	Not available

IHH, immunohistochemistry; POD, peroxidase.

Cells grown on glass coverslips were stained by the same procedure used for wax sections (excluding dewaxing and antigen retrieval) but were labelled with biotinylated secondary antibodies, and then, peroxidase-conjugated streptavidin and staining was visualized by the 3,3′-diaminobenzidine peroxidase reaction with nickel enhancement (**Table 1**). After 3,3′-diaminobenzidine/H_2_O_2_/nickel development, cells were thoroughly washed with water and the coverslips were removed from the wells and mounted on microscope slides with Hydromount^™^ (National Diagnostics). A detailed protocol is available via this link: dx.doi.org/10.17504/protocols.io.kqdg39yjpg25/v1.

Images were obtained using an Olympus BX43 microscope and AKOYA Biosciences Mantra II multispectral imaging system.

### Real-time quantitative PCR

Cells were processed to extract total RNA and protein using TRI Reagent® solution (Thermo Fisher Scientific, AM9738). Total RNA was quantified using a NanoDrop 1000 spectrophotometer (Thermo Fisher Scientific) and cDNA generated from 2 µg using a high-capacity cDNA reverse transcription kit and random hexamer primers (Thermo Fisher Scientific, 4368814). Quantitative real-time PCR was conducted with a StepOne Real-Time PCR System (Applied Biosystems) using PowerUp^™^ SYBR^™^ Green Master Mix (Thermo Fisher Scientific, A25742) and gene-specific (BLASTN, RRID:SCR_001598) primers (Table 1) to quantify relative gene expression levels of α-synuclein and TNF mRNA in the samples normalized to β-actin using the comparative 2^−ΔΔ^C_T_ method.^45^ A detailed protocol is available via this link: dx.doi.org/10.17504/protocols.io.rm7vzb2z2vx1/v1.

### Western blot

Protein pellets from the TRI Reagent® extraction were dissolved in 2% SDS containing 8 M urea or cells were lysed in radioimmunoprecipitation assay (RIPA) buffer (Merck, R0278) containing protease inhibitors (Merck, P0044). Samples (∼10 µg total protein) were separated by polyacrylamide gel electrophoresis using Bolt^™^ or NuPAGE^™^ 12%, Bis-Tris, 1.0 mm, Mini Protein Gels (Thermo Fisher Scientific, NW00127BOX or NP0349BOX). Following electrophoresis, protein was transferred onto polyvinylidene difluoride (PVDF) membranes (Thermo Fisher Scientific, 88520). Membranes were then rinsed with PBS, fixed for 20 min with 4% paraformaldehyde in PBS and then washed (4 × 5 min) with PBST [PBS containing 0.01% (*v*/*v*) Tween^™^-20]. Membranes were then incubated with block solution (PBST containing 2% (*w*/*v*) bovine serum albumin (BSA) and 0.005% (*w*/*v*) thiomersal for 1 h and were then incubated with rabbit monoclonal antibodies against α-synuclein and β-actin overnight diluted in block solution (Table 1). Protein bands were visualized by 3,3′-diaminobenzidine/peroxidase reaction or with enhanced chemiluminescence (ECL) plus reagents (Merck, GERPN2232), digitized with a ChemiDoc^™^ MP imaging system (Biorad) and analysed using ImageJ v1.53u (RRID:SCR_003070, https://imagej.net/). A detailed protocol is available via this link: dx.doi.org/10.17504/protocols.io.bp2l69pqdlqe/v1.

### Enzyme-linked immunosorbent assay

The amount of α-synuclein in the culture medium was measured using a SimpleStep ELISA® mouse α-synuclein colourmetric sandwich enzyme-linked immunosorbent assay (ELISA) kit (Abcam, ab282865) according to the manufacturer’s instructions. Briefly, an aliquot of culture medium was incubated with a cocktail of capture and detecting antibodies for 2 h. After washing, 3,3′,5,5′-tetramethylbenzidine (TMB) substrate was added. Once developed, the reaction was stopped with acid stop solution and the absorption at 450 nm was measured with a Cytation 5 imaging multimode reader.

### Proteosome assay

Ubiquitin-proteasome system (UPS) activation was assessed using a proteasome activity assay kit (Abcam ab107921) according to the manufacturer’s instructions. Briefly, an aliquot of cell lysate was mixed with proteosome inhibitor and substrate and incubated at 37°C for 25 min followed by measuring output (T_1_) on a Cytation 5 imaging multimode reader (Ex/Em = 350/440 nm). Samples were then incubated for a further 15 min at 37°C and then output (T_2_) was measured.

### Data analysis

Data were analysed and visualized with GraphPad Prism^™^ v.9.5 (RRID:SCR_002798, http://www.graphpad.com/). Parametric data (Shapiro–Wilk, *P* > 0.05) were analysed by one-way ANOVA (*F*) and non-parametric data by Kruskal–Wallis one-way ANOVA by ranks (*H*). When ANOVA indicated a significant difference, *post hoc* multiple comparison pair-wise comparisons of all treatment groups or multiple comparisons of all treatment groups to the control group were made. Values are given as mean ± standard error of the mean. All tests were two-tailed and the significance level for all tests was taken to be *P* < 0.05.

## Results

### Enteroendocrine cells but not enterocytes express α-synuclein

**Figure 1:**
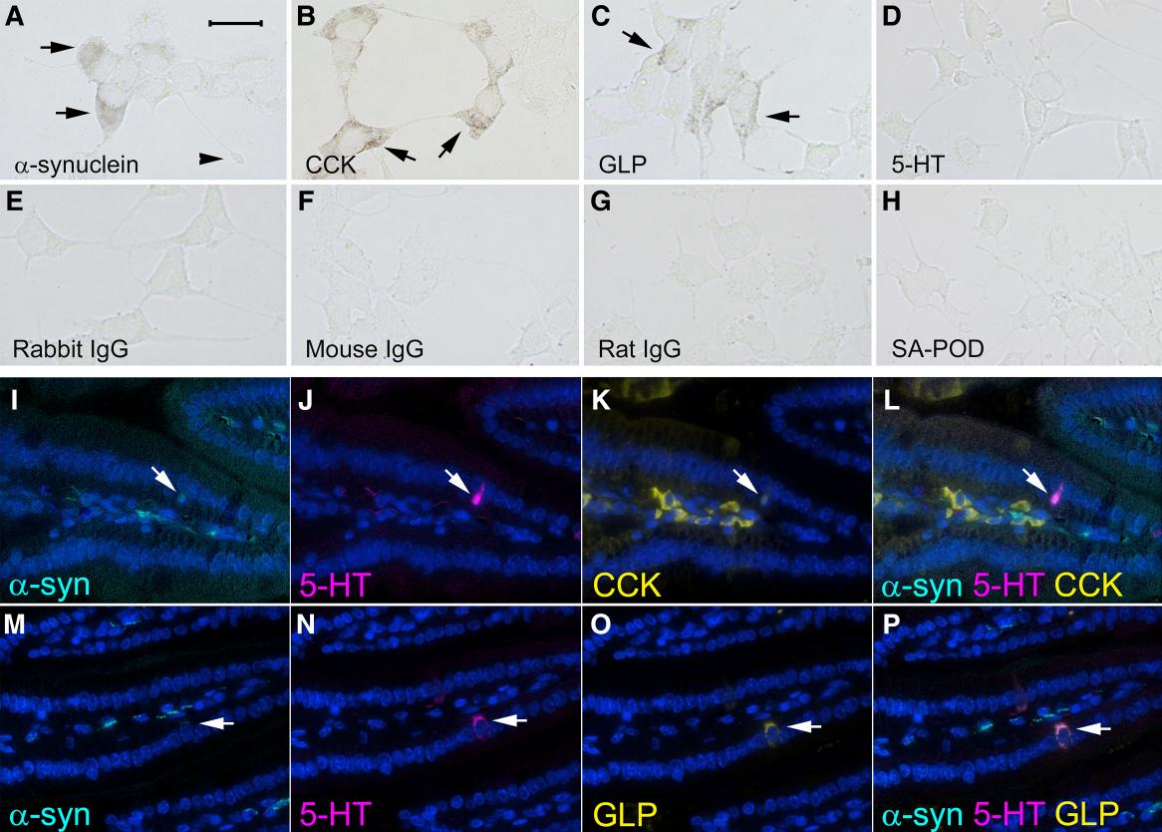
**Representative images showing α-synuclein and markers of enteroendocrine cells examined by immunohistochemistry in STC-1 cells (A–H) and by immunofluorescence in longitudinal sections of mouse ileum (I–P).** Immunoreactivity for (**A**) α-synuclein (arrows), (the arrowhead shows a neuropod), (**B**) CCK (arrows) and (**C**) GLP (arrows). (**D**) 5-HT staining was not evident in STC-1 cells. No immunoreactivity was detected when primary antibodies were omitted and just biotinylated (**E**) rabbit, (**F**) mouse or (**G**) rat immunoglobulins and streptavidin conjugated with peroxidase (SA-POD) were used or (**H**) just SA-POD was used. Immunofluorescence for (**I** and **M**) α-synuclein (arrow), (**J** and **N**) 5-HT (arrow), (**K**) CCK (arrow) and (**O**) GLP (arrow). (**L**) Co-expression of 5-HT, CCK and α-synuclein (arrow). (**P**) Co-expression of 5-HT, GLP and α-synuclein (arrow). Cellular nuclei were stained with DAPI. The bar in **A** = 25 μm for **A**–**H** and the bar in **I** = 100 μm for **I–P**.

Diffuse α-synuclein immunoreactivity and punctate cholecystokinin (CCK) and glucagon-like peptide-1 (GLP) immunoreactivity was detected in STC-1 cells, but staining for 5-hydroxytryptamine (5-HT) was not detectable (Fig. 1A–D). CCK and GLP are both released by and indicative of enteroendocrine cells.

No staining was observed when primary antibodies were omitted, and just biotinylated secondary antibody and streptavidin–peroxidase were used for the immunohistochemistry procedure (Fig. 1E–G). No staining was seen when just streptavidin–peroxidase alone was incubated with the cells (Fig. 1H).

Enteroendocrine cells within the epithelium of mouse ileum and central neural tissue of villi expressed α-synuclein (Fig. 1I and M) with higher levels present in neural tissue compared with the enteroendocrine cells. CCK, GLP and 5-HT immunofluorescence was detected in enteroendocrine cells, whereas the enteroendocrine cell markers and α-synuclein were not detectable in ileal enterocytes (Fig. 1I–P). CCK was also present in stromal cells of the villi (Fig. 1K).

### Effect of TLR and FFA2/3 agonists on α-synuclein

**Figure 2:**
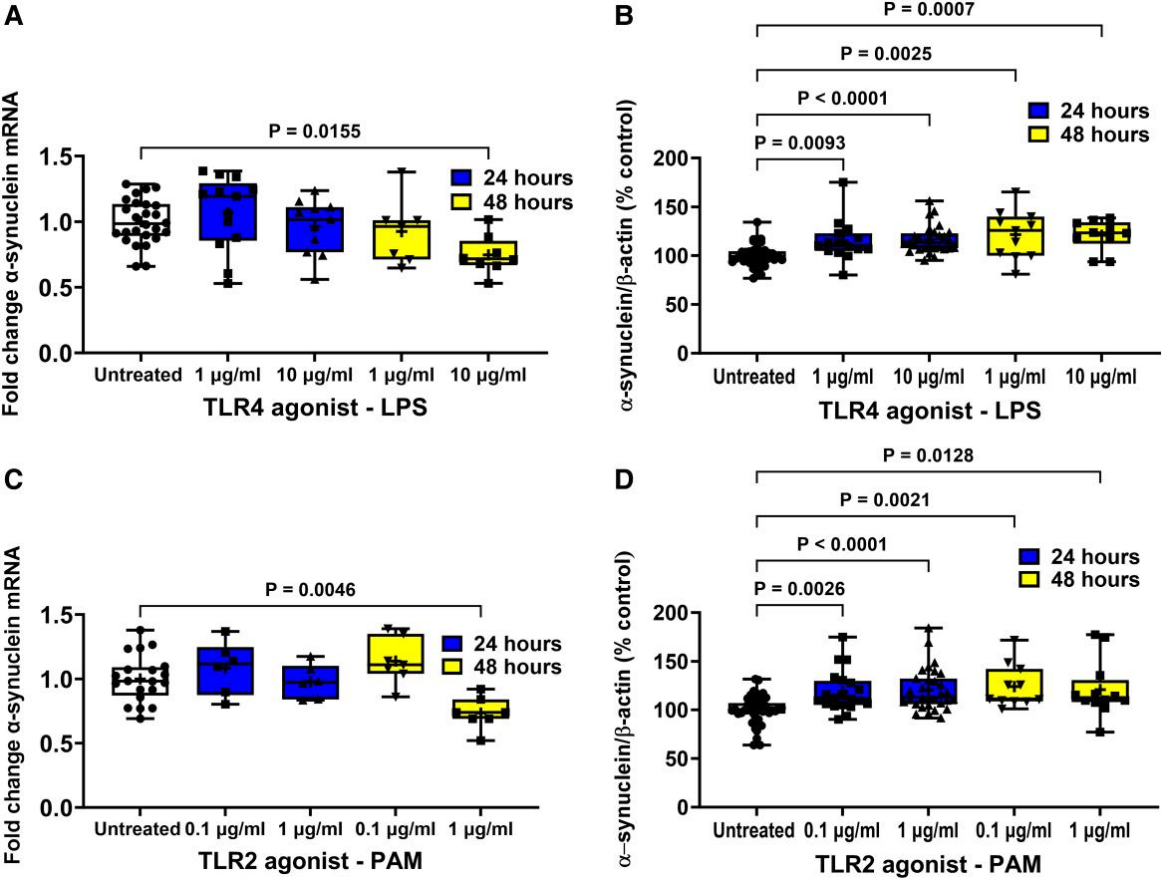
**Effect of TLR agonists on α-synuclein expression in STC-1 cells.** LPS and PAM treatment of STC-1 cells decreased the level of α-synuclein mRNA after 48 h but increased the level of intracellular α-synuclein protein. SCFA had opposing effects on α-synuclein mRNA and protein. (**A**) After 48 hours 10 μg/ml LPS caused a significant reduction in α-synuclein mRNA. (**B**) Treatment for 24 and 48 h with LPS caused a significant increase in intracellular α-synuclein (**C**) After 48 hours 1 μg/ml PAM caused a significant reduction in α-synuclein mRNA. (**D**) Treatment for 24 and 48 h with PAM caused a significant increase in intracellular α-synuclein. Data are presented as box (25th and 75th percentiles) and whisker (5th and 95th percentiles), median (line) and mean (+) with all data points shown. For qPCR, each data point represents triplicate or duplicate analysis of total RNA extracted from a well of a 12-well cell culture plate expressed as fold change of α-synuclein mRNA compared with untreated cells normalized to β-actin using the 2^−ΔΔ^C_T_ method. For western blot, each data point represents protein extracted from a well of a 12-well cell culture plate expressed as relative levels of α-synuclein in comparison with untreated cells normalized to β-actin. Data were analysed by one-way ANOVA and *post hoc* Dunnett’s or Dunn’s multiple comparison test.

**Figure 3:**
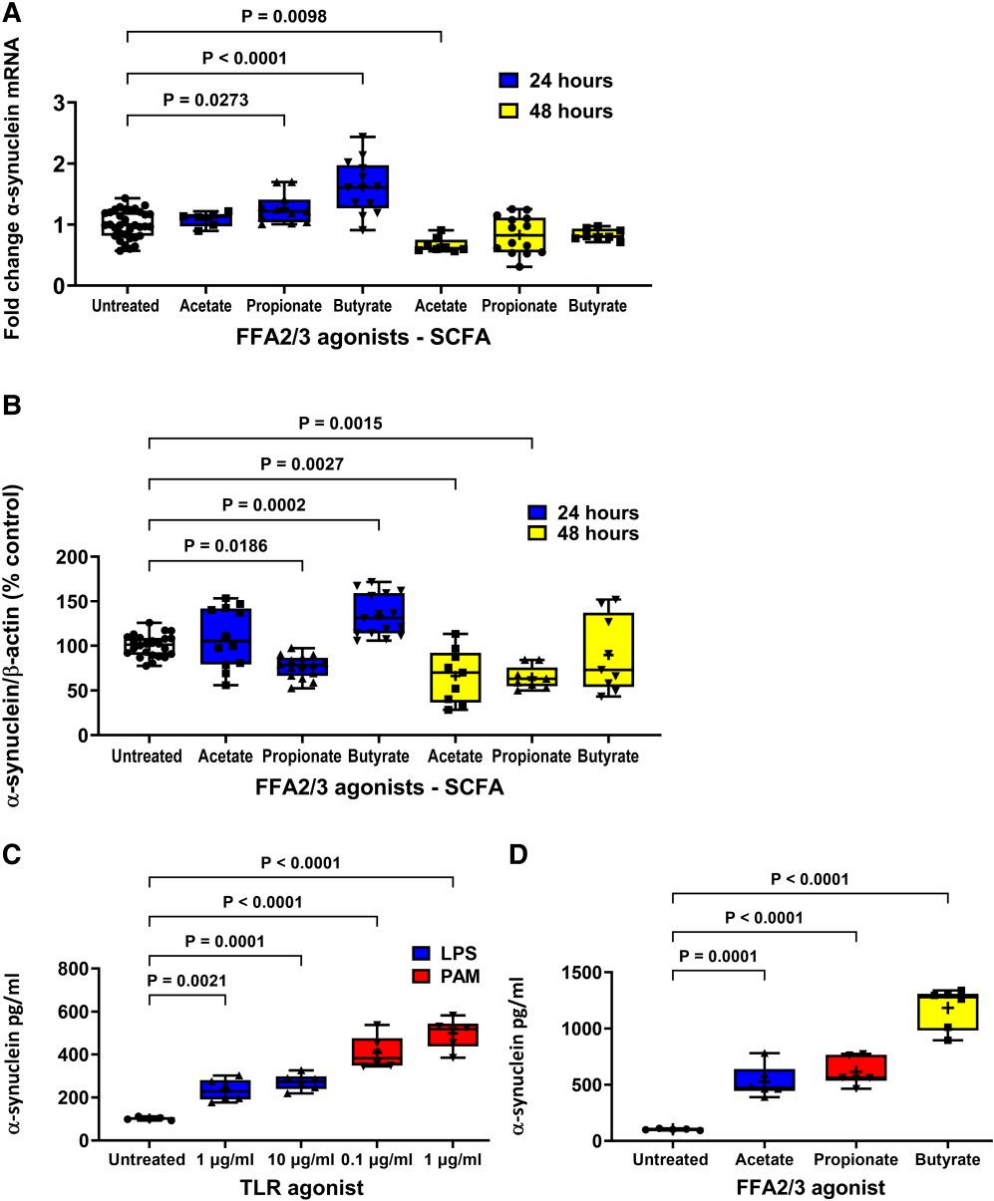
**Effect of FFA2/3 agonists on α-synuclein expression and TLR and FFA2/3 agonists on α-synuclein release from STC-1 cells.** (**A**) After 24 hours 2 mM butyrate and 4 mM propionate increased α-synuclein mRNA and 4 mM acetate reduced α-synuclein mRNA after 48 h. (**B**) After 24 h 2 mM butyrate increased intracellular α-synuclein, whereas 4 mM propionate decreased intracellular α-synuclein. After 48 h, 4 mM acetate and 4 mM propionate caused a reduction in intracellular α-synuclein. TLR (**C**) and FFA2/3 (**D**) agonists increased release of α-synuclein from STC-1 cells. Data are presented as box (25th and 75th percentiles) and whisker (5th and 95th percentiles), median (line) and mean (+) with all data points shown. For qPCR, each data point represents triplicate or duplicate analysis of total RNA extracted from a well of a 12-well cell culture plate expressed as fold change of α-synuclein mRNA compared with untreated cells normalized to β-actin using the 2^−ΔΔ^C_T_ method. For western blot, each data point represents protein extracted from a well of a 12-well cell culture plate expressed as relative levels of α-synuclein in comparison with untreated cells normalized to β-actin. For ELISA, each data represents an aliquot of medium taken from a well of a 12-well cell culture plate expressed as fold change of α-synuclein compared with untreated cells. Data were analysed by one-way ANOVA and *post hoc* Dunnett’s or Dunn’s multiple comparison test.

STC-1 cells constitutively express and release α-synuclein and this was augmented by TLR and FFA2/3 agonists (Figs. 2 and 3). Initial experiments sought to determine the effect of stimulation of TLR and FFA2/3 on α-synuclein mRNA measured by qPCR, intracellular α-synuclein levels measured by western blot and release of α-synuclein into the cell culture medium measured by ELISA. Treatment with TLR agonists had no effect on cell viability (Supplementary Fig. 1). A representative western blot is shown in Supplementary Fig. 2. Data for each graph and images of all western blots can be found at the Zenodo data repository (https://doi.org/10.5281/zenodo.7712730).

### α-Synuclein expression

Stimulation of STC-1 cells with 1 and 10 μg/ml LPS had no effect on the level of α-synuclein mRNA in comparison with untreated cells in cells treated for 6 (data not shown) or 24 h, but after 48 h, the 10 μg/ml concentration of LPS caused a 25% reduction [*F*(4,57) = 4.168, *P* = 0.0155] in α-synuclein mRNA compared with untreated cells after 48 h (Fig. 2A).

Stimulation of STC-1 cells with 1 and 10 μg/ml LPS significantly increased [*H*(4,101) = 14.29] intracellular α-synuclein by 15% (*P* = 0.0093) and 17% (*P* < 0.0001), respectively, after 24 h and by 22% (*P* = 0.0025) and 18% (*P* = 0.0007), respectively, after 48 h in comparison with untreated cells (Fig. 2B).

Stimulation of STC-1 cells with 0.1 and 1 μg/ml PAM had no effect after 6 (data not shown) or 24 h, but after 48 h, the higher concentration caused a significant 26% reduction [*F*(4,40) = 6.143, *P* = 0.0046] in the level of α-synuclein mRNA compared with untreated cells (Fig. 2C).

Stimulation of STC-1 cells with 0.1 and 1 μg/ml PAM significantly increased [*F*(4,99) = 9.621] the level of intracellular α-synuclein by 16% (*P* = 0.0026) and 19% (*P* < 0.0001), respectively, after 24 h and by 40% (*P* = 0.0021) and 28% (*P* = 0.0128), respectively, after 48 h treatment in comparison with untreated cells (Fig. 2D).

Stimulation of STC-1 cells with 2 mM butyrate and 4 mM propionate for 24 h significantly increased [*F*(6,90) = 22.04] the level of α-synuclein mRNA by 57% (*P* < 0.0001) and 26% (*P* = 0.0273), respectively, while stimulation with 4 mM acetate had no effect. In contrast, after 48 h, butyrate and propionate had no effect on α-synuclein mRNA, whereas acetate caused a significant reduction of 36% (*P* = 0.0098) compared with untreated cells (Fig. 3A).

Stimulation of cells for 24 h with butyrate increased [*F*(6,125) 23.03] intracellular α-synuclein by 35% (*P* < 0.0002), whereas propionate decreased intracellular α-synuclein by 31% (*P* < 0.0186). After 48 h, acetate and propionate caused a 34% (*P* = 0.0028) and 32% (*P* = 0.0015) reduction in intracellular α-synuclein, respectively. TLR and FFA2/3 agonists increased release of α-synuclein from STC-1 cells. Stimulation of cells with butyrate and propionate for 24 h significantly increased and decreased, respectively, the level of intracellular α-synuclein, while acetate had no effect. In contrast, after 48 h, butyrate had no effect on the level of α-synuclein, whereas acetate and propionate caused a significant reduction (*P* = 0.0001) (Fig. 3B).

### α-Synuclein release

Untreated STC-1 cells released α-synuclein into culture medium that resulted in a concentration of 110 ± 1 pg/ml in a 24 h period. Stimulation of STC-1 cells with 1 and 10 μg/ml LPS for 24 h caused a significant increase [*H*(2,15) = 12.12] in the amount of released α-synuclein to 230 ± 5 (*P* = 0.0021) and 270 ± 2 pg/ml (*P* < 0.0001), respectively (Fig. 3C). Stimulation of STC-1 cells with 0.1 and 1 μg/ml PAM for 24 h caused a significant increase [*H*(2, 15) = 12.78] in the amount of released α-synuclein to 410 ± 3 (*P* = 0.0001) and 500 ± 3 pg/ml (*P* < 0.0001), respectively (Fig. 3C).

Stimulation of cells with acetate, propionate or butyrate for 24 h caused a significant increase [*F*(3,19) = 60.44] in the amount of released α- synuclein to 530 ± 6 (*P* = 0.0001), 610 ± 5 (*P* < 0.0001) and 1180 ± 8 pg/ml (*P* < 0.0001), respectively (Fig. 3D).

### TLR antagonists reduce agonist-induced α-synuclein accumulation and release

**Figure 4:**
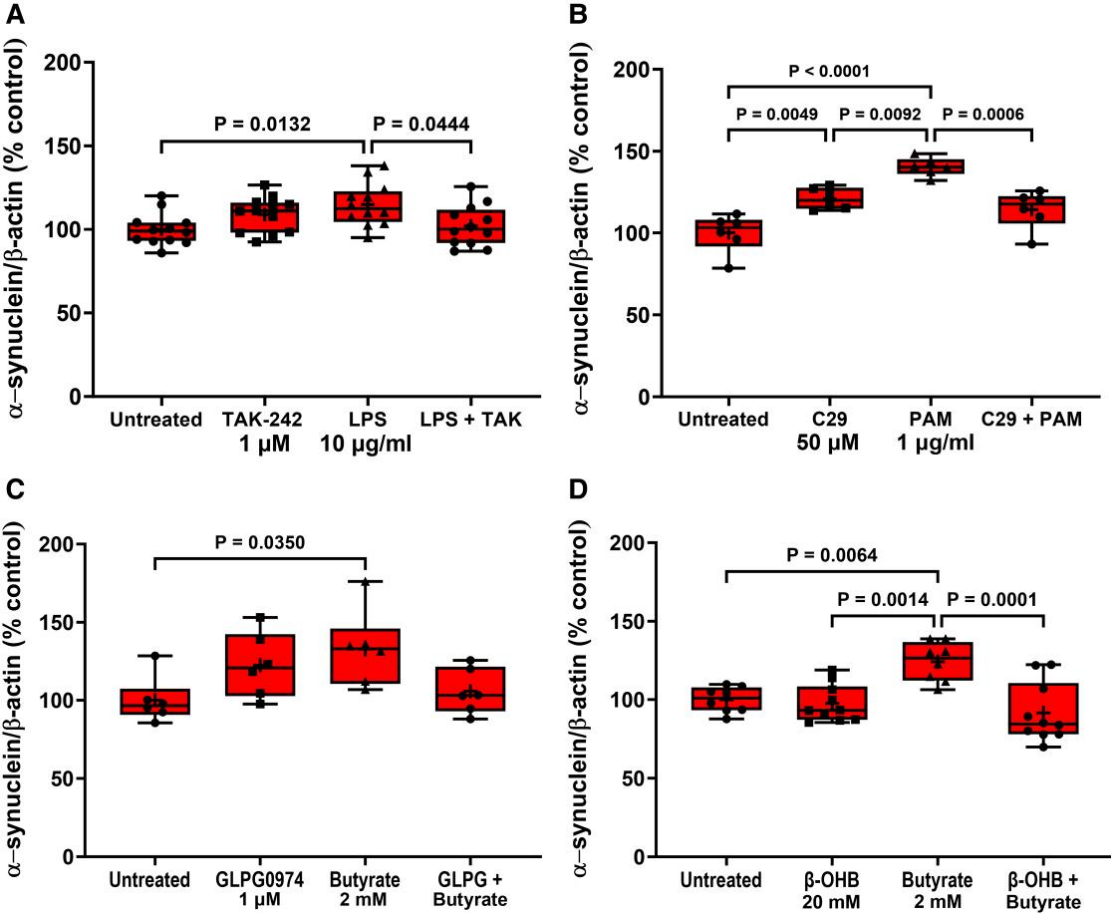
**Antagonists prevent agonist-induced STC-1 cell α-synuclein accumulation.** Antagonists selective for TLR4, TLR2, FFA2 and FFA3 receptors significantly reduced the effect of 24 h treatment of STC-1 cells with their respective agonists on intracellular α-synuclein content. (**A**) TAK-242 (1 μM) reduced LPS-induced increase in intracellular α-synuclein. (**B**) C29 (50 μM) reduced the PAM-induced increase in intracellular α-synuclein. (**C**) GLPG0974 (1 μM) reduced the butyrate-induced increase in intracellular α-synuclein. (**D**) β-hydroxybutyrate (20 mM) reduced the butyrate-induced increase in intracellular α-synuclein. Each data point represents protein extracted from a well of a 12-well cell culture plate expressed as relative levels of α-synuclein in comparison with untreated cells normalized to β-actin. Data are presented as box (25th and 75th percentiles) and whisker (5th and 95th percentiles), median (line) and mean (+) with all data points shown and were analysed by one-way ANOVA and *post hoc* Tukey’s multiple comparison test.

**Figure 5:**
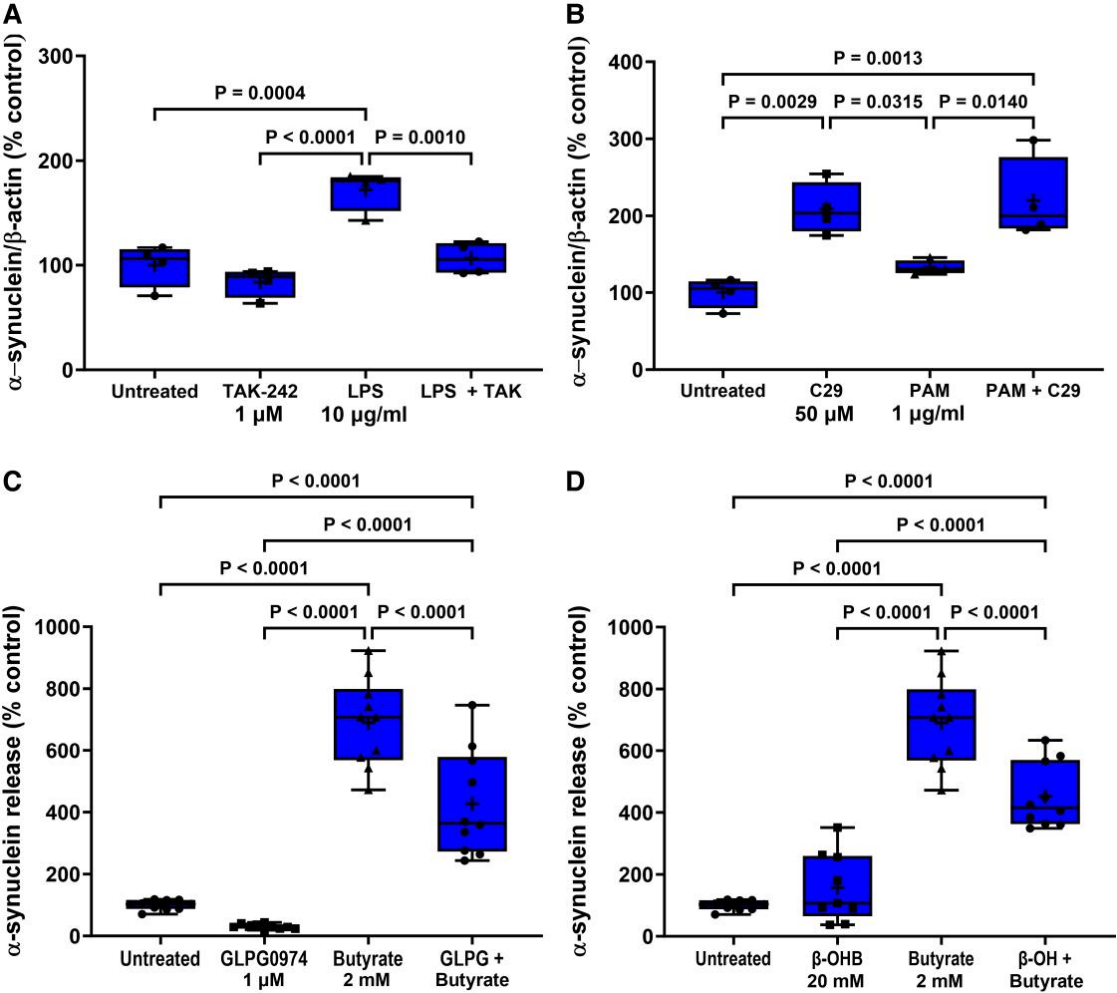
**Antagonists prevent agonist-induced STC-1 cell α-synuclein release.** Antagonists selective for TLR4, TLR2, FFA2 and FFA3 receptors significantly reduced the effect of 24 h treatment of STC-1 cells with their respective agonists on α-synuclein release. (**A**) TAK-242 (1 μM) reduced the LPS-induced increase in α-synuclein release. (**B**) C29 (50 μM) alone increased α-synuclein release and was unaffected by PAM. (**C**) GLPG0974 (1 μM) reduced the butyrate-induced increase in α-synuclein release. (**D**) β-hydroxybutyrate (20 mM) reduced the butyrate-induced increase in α-synuclein release. Each data point represents analysis of an aliquot of medium taken from a well of a 12-well cell culture plate expressed as fold change of α-synuclein compared with untreated cells. Data are presented as box (25th and 75th percentiles) and whisker (5th and 95th percentiles), median (line) and mean (+) with all data points shown and were analysed by one-way ANOVA and *post hoc* Tukey’s multiple comparison test.

Selective antagonists were used to demonstrate that agonist effects were receptor-mediated and not due to non-specific actions. The higher concentration of each TLR agonist was chosen because these gave the greatest and most robust response. Stimulation of STC-1 cells with 10 μg/ml LPS for 24 h significantly increased intracellular α-synuclein by 15% (*P* = 0.0132). Pre-treatment for 1 h with TAK-242 (1 µM) significantly reduced (*P* = 0.0444) the LPS-induced increase in intracellular α-synuclein. Stimulation of STC-1 cells with LPS for 24 h significantly increased α-synuclein release by 75% (*P* = 0.0004), and this was significantly reduced (*P* = 0.0010) by 1 μM TAK-242. TAK-242 treatment alone did not alter the intracellular α-synuclein level or release of α-synuclein (Figs. 4A and 5A).

Similarly, pre-treatment for 1 h with C29 (50 µM) reduced the increase in intracellular α-synuclein [*F*(3,20) = 19.13, *P* < 0.0001] induced by 1 μg/ml PAM after 24 h but had no effect on release. However, C29 treatment alone increased intracellular α-synuclein [*F*(3,20) = 19.13, *P* < 0.0049] and release [*F*(3,12) = 12.24, *P* = 0.0029]. PAM did not cause significant α-synuclein release in this experiment when analysed with Tukey’s multiple comparison test; however, the difference between untreated and PAM treated cells was significant when analysed by an unpaired two-tailed *t*-test [*t* = 3.087, df = 6, *P* = 0.0215] (Figs. 4B and 5B).

### FFA2/3 antagonists reduce butyrate-induced α-synuclein accumulation and release

Stimulation of STC-1 cells with 2 mM butyrate for 24 h increased intracellular α-synuclein by 33% (*P* = 0.0350) in comparison with untreated cells, and this was mitigated by pre-treatment of the STC-1 cells for 1 h with the FFA2 antagonist GLPG0974 (1 µM). Stimulation of STC-1 cells with butyrate for 24 h increased release [*F*(3,36) = 75.48, *P* < 0.0001] of α-synuclein by 690% (*P* < 0.0001), and this was significantly reduced (*P* < 0.0001) by GLPG0974 (1 µM). GLPG0974 treatment alone had no significant effect on intracellular α-synuclein levels or release (Figs. 4C and 5C).

Stimulation of STC-1 cells with 2 mM butyrate for 24 h significantly increased intracellular α-synuclein by 24% (*P* = 0.0064) in comparison with untreated cells, and this was significantly reduced (*P* = 0.0001) by pre-treatment for 1 h of STC-1 cells with the FFA3 antagonist β-hydroxybutyrate (20 mM). Stimulation of STC-1 cells with butyrate for 24 h increased release by 690% (*P* = 0.0001), and this was significantly reduced (*P* = 0.0001) by pre-treatment of STC-1 cells with the FFA3 antagonist β-hydroxybutyrate (20 mM) (Figs. 4D and 5D).

### TLR and FFA2/3 agonists stimulate TNF mRNA production

**Figure 6:**
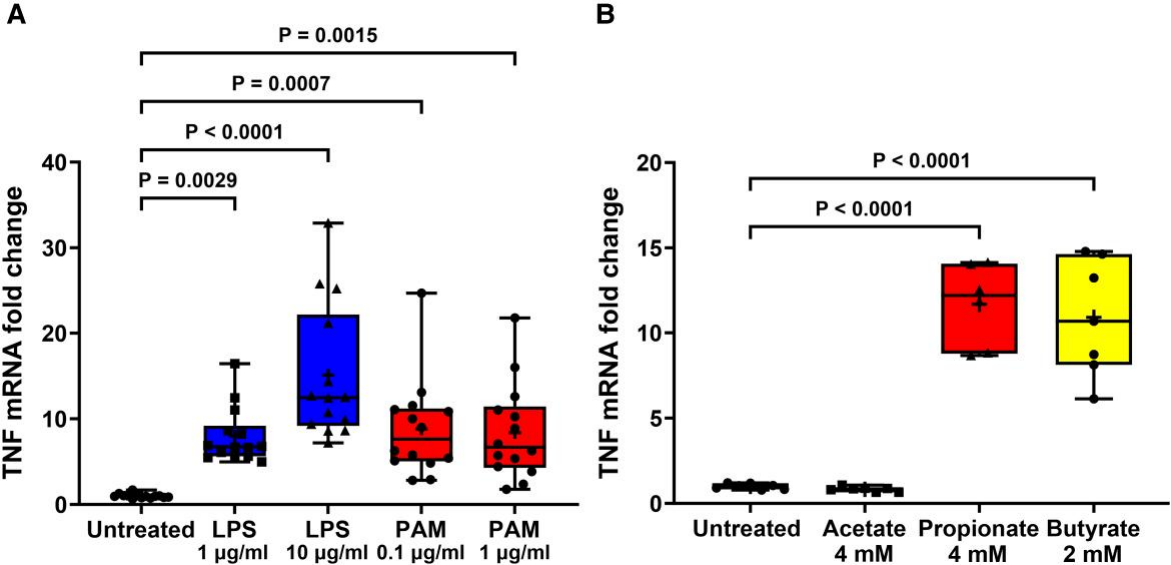
**TLR and FFA2/3 agonists increase TNF transcription in STC-1 cells.** LPS, PAM and SCFA treatment of STC-1 cells significantly increased the amount of TNF mRNA. (**A**) Stimulation of STC-1 cells for 24 h with LPS or PAM increased TNF mRNA. (**B**) Stimulation of STC-1 cells with 4 mM acetate had no effect on TNF mRNA, whereas 4 mM propionate and 2 mM butyrate increased TNF mRNA. Data are presented as box (25th and 75th percentiles) and whisker (5th and 95th percentiles), median (line) and mean (+) with all data points shown. Each data point represents triplicate or duplicate analysis of total RNA extracted from a well of a 12-well cell culture plate expressed as fold change of TNF mRNA compared with untreated cells normalized to β-actin using the 2^−ΔΔ^C_T_ method. Data were analysed by one-way ANOVA and *post hoc* Tukey’s multiple comparison test.

The effect of TLR and FFA2/3 activation on TNF mRNA transcription in STC-1 cells was investigated. Stimulation of STC-1 cells for 24 h with 1 and 10 μg/ml LPS for 24 h increased [*H*(4,65) = 40.54] TNF mRNA by 6.9-fold (*P* = 0.0029) and 16.2-fold (*P* < 0.0001), respectively, and stimulation of cells with 0.1 and 1 μg/ml PAM for 24 h increased TNF mRNA by 7.9-fold (*P* = 0.0007) and 7.8-fold (*P* = 0.0015), respectively, in comparison with untreated cells (Fig. 6A). Stimulation of STC-1 cells with acetate had no effect on TNF mRNA, whereas propionate and butyrate increased [*F*(3,23) = 55.69] TNF mRNA by 11.7-fold (*P* < 0.0001) and 12.2-fold (*P* < 0.0001), respectively, in comparison with untreated cells (Fig. 6B).

**Figure 7:**
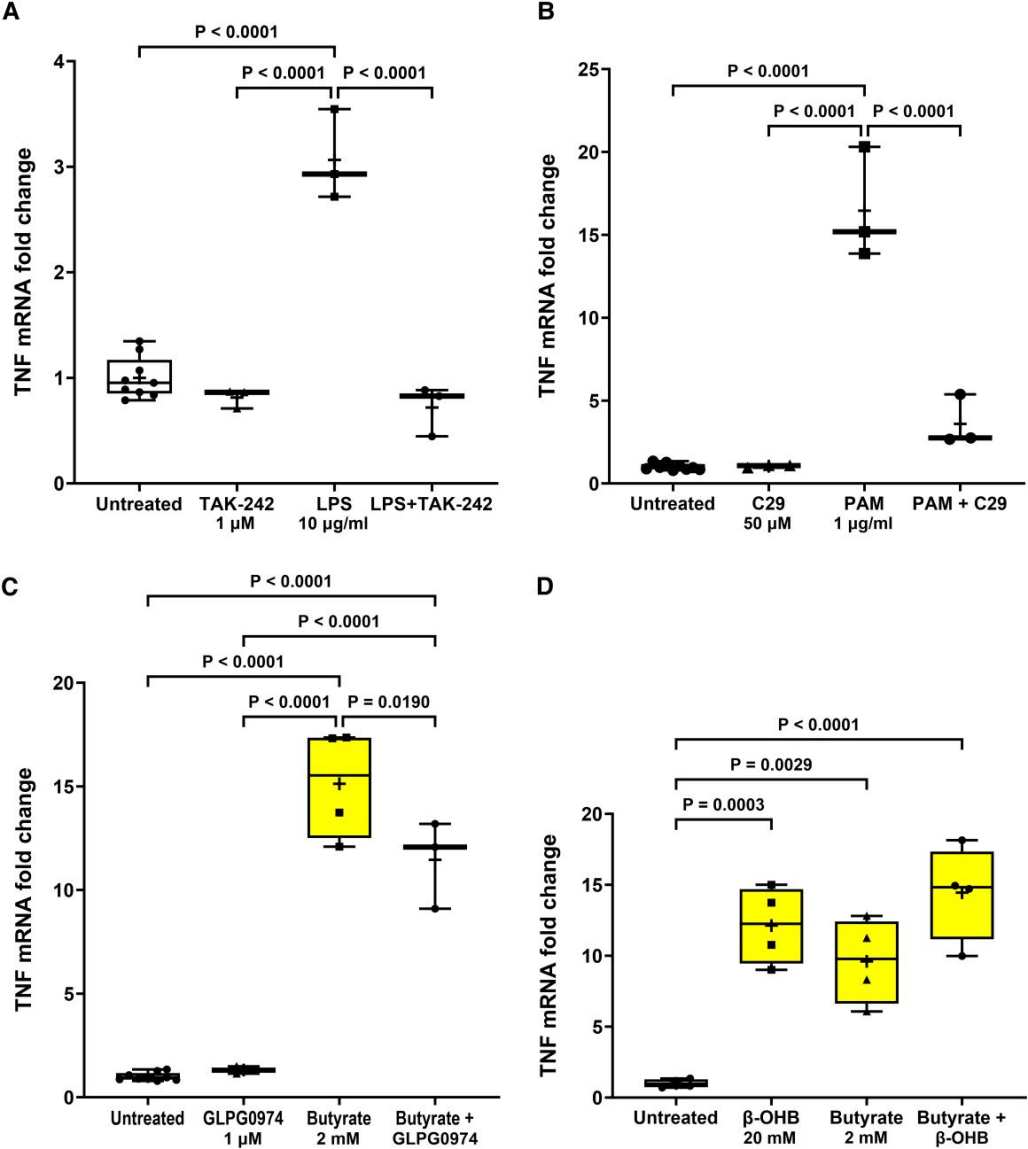
**TLR and FFA2/3 antagonists prevent agonist-induced TNF transcription in STC-1 cells.** Pre-treatment for 1 h with (**A**) TAK-242 (1 µM) significantly reduced the LPS-induced increase in TNF mRNA, (**B**) C29 (50 µM) significantly reduced the PAM-induced increase in TNF mRNA, (**C**) GLPG0974 (1 µM) significantly reduced the butyrate-induced increase in TNF mRNA, and (**D**) β-hydroxybutyrate (β-OHB) (20 mM) had no effect on the butyrate-induced increase in TNF mRNA. Data are presented as box (25th and 75th percentiles) and whisker (5th and 95th percentiles), median (line) and mean (+) with all data points shown. Each data point represents triplicate or duplicate analysis of total RNA extracted from a well of a 12-well cell culture plate expressed as fold change of TNF mRNA compared with untreated cells normalized to β-actin using the 2^−ΔΔ^C_T_ method. Data were analysed by one-way ANOVA and *post hoc* Tukey’s multiple comparison test.

The induction of TNF mRNA following LPS, PAM and butyrate exposure for 24 h was significantly reduced by TAK-242 [*F*(3,14) 68.72, *P* < 0.0001], C29 [*H*(3,14) = 12.16, *P* = 0.0003] and GLPG0974 [*F*(3,15) = 117.6, *P* < 0.0001], respectively, confirming specific activation of TLR4, TLR2 and FFA2 and involvement of these receptors in stimulation of TNF transcription (Fig. 7A–C). β-Hydroxybutyrate did not affect butyrate-induced TNF transcription, suggesting that the action of butyrate on TNF mRNA transcription was not through FFA3 receptors. However, β-hydroxybutyrate alone caused a significant increase in TNF mRNA [*F*(3,12) = 19.74, *P* < 0.0001] (Fig. 7D).

### Ubiquitin-proteosome system

The UPS pathway was evaluated in cells stimulated with LPS 1 μg/ml or PAM 0.1 μg/ml for 24 and 48 h. Compared with untreated cells, there was a significant increase in proteosome activity in cells stimulated with either LPS or PAM at both time points [*F*(4,13) = 21.93, *P* < 0.001] (Supplementary Fig. 3), indicating that UPS impairment was not responsible for α-synuclein accumulation upon TLR activation.

## Discussion

The hypothesis that structural bacterial components and/or bacterial metabolites, such as SCFA, could alter α-synuclein expression in enteroendocrine cells was tested. Stimulation of enteroendocrine cells with TLR2 and TLR4 agonists, a lipopeptide and LPS, respectively, increased the level of intracellular α-synuclein and release of α-synuclein into the culture medium, and this effect was reduced by TLR antagonists. Activation of enteroendocrine cells with SCFA, particularly butyrate, increased release of α-synuclein into the culture medium. Again, the effect of butyrate was reduced by FFA2 and FFA3 receptor antagonists. Interestingly, butyrate and propionate had an opposing effect on intracellular α-synuclein levels at the 24 h time point, possibly due to the different affinities of each SCFA for the FFA2 and 3 receptors, the additional action of butyrate as a histone deacetylase inhibitor or other non-FFA receptor actions.^46^ Selective FFA receptor agonists or the histone deacetylase inhibitor trichostatin A could be used to investigate this further. Overall, these results suggest that enteroendocrine cells could be a site of α-synuclein accumulation in Parkinson’s disease when stimulated by specific bacterial ligands through TLR2/4 activation and butyrate acting on FFA2/3.

It has been proposed that the pathological changes in a ‘body-first’ Parkinson’s disease subtype might begin within the gastrointestinal tract. However, the factors that can initiate these changes and where in the gastrointestinal tract they might occur are unknown.^10^ Exposure to Gram-negative and Gram-positive bacterial components, such as LPS and lipopeptides (PAM), increased intracellular accumulation and release of α-synuclein. The observed changes seemed to be specific to TLR4 and TLR2 activation because of the effective inhibition of LPS- and PAM-induced increase in intracellular α-synuclein and release upon treatment with their respective antagonists. Activation of TLR4 with LPS and TLR2 with PAM resulted in the production of mRNA for the inflammatory cytokine TNF. Again, this effect on TNF was significantly reduced using selective antagonists. Butyrate also caused a large increase in TNF mRNA, and this effect was not completely abolished by GLPG0974 (or β-hydroxybutyrate) suggestive of an additional mechanism to FFA activation for the induction of TNF mRNA by butyrate.

Bacterial lipopeptides are present in almost all Gram-positive bacteria and recognized by the TLR2 of the innate immune system. Amongst others, they play a central role in immune response and pathogenicity as bacterial species establish their ecological niche as commensals through their lipid structures.^47^ The role of TLR2 in Parkinson’s disease pathogenesis has been recently evaluated in several *in vivo* and *in vitro* models. Post-mortem studies have shown increased levels of TLR2 protein in the brain from patients with Parkinson’s disease when compared with age-matched controls, with a positive correlation with disease status and duration.^48^ Knocking out TLR2 in transgenic mice expressing human A53T α-synuclein reduced levels of α-synuclein.^33^  *In vitro*, differentiated SHSY5Y transduced with lentiviral -synuclein and treated with PAM showed increased levels of insoluble α-synuclein oligomers.^33^ Moreover, oligomeric forms of neuron-released α-synuclein activated microglial TLR2.^49^ Interestingly, small molecules inhibiting the TLR signal pathway ameliorated the PAM-induced increase of both TLR2 and α-synuclein in differentiated SHSY5Y cells, suggesting a possible therapeutic application of TLR2-targeting agents in Parkinson’s disease.^48^ For the first time, we have analysed the role of TLR2 pathway in gut enteroendocrine cells and provided evidence supporting the link between TLR2 and Parkinson’s disease pathogenesis in response to bacterial components. Application of TLR2 inhibitors or antagonists is of interest to mitigate pathogenic changes in the gut. The impact of TLR2 inhibitors should, however, be carefully considered in relation to those Gram-positive bacteria within the phylum *Firmicutes* which are particularly rich in lipopeptides. Among those, there is *Enterococcus faecalis*, an opportunistic pathogen responsible for nosocomial infections,^50^ and *E. faecalis* is one of the key bacteria affecting levodopa bioavailability.^51^ For these reasons, within Parkinson’s disease, suppressing host TLR activity requires careful consideration not only for the well-known increased vulnerability to infection^52^ but also for the potential impact on certain bacterial niches and its implication on disease course.

Neuroinflammation is implicated in Parkinson’s disease pathogenesis, and several models have used LPS to mimic the inflammatory events seen in Parkinson’s disease.^53^ Patients with Parkinson’s disease have increased expression of TLR4, enhanced markers of bacterial translocation and higher proinflammatory gene profiles in the colon when compared with healthy controls.^32^ In TLR4 knockout mice, rotenone induced less intestinal inflammation, neuroinflammation and motor dysfunction compared with wild-type animals.^32^ Could treatments modulating the TLR4 inflammatory response ameliorate Parkinson’s disease? In our study, TAK-242, which is already under investigation in liver disease (ClinicalTrials.gov ID: NCT04620148), reduced the LPS-induced accumulation of α-synuclein in the enteroendocrine cells and testing such drugs in LPS models of Parkinson’s disease is warranted.

Recent studies evaluating the gut microbiome in Parkinson’s disease patients found an increase of opportunistic pathogens (*Porphyromonas*, *Prevotella*, *Corynebacterium 1*).^54^ Hence, we can hypothesize that these commensal bacteria could become prevalent in certain individuals and thus induce pathological changes seen in Parkinson’s disease. *In vitro* modelling should test this hypothesis.

The same study also found a reduction of SCFA-producing bacteria in the Parkinson’s disease population,^54^ which has been confirmed in other patient cohorts with reduced levels of SCFA detected in stool samples.^37^ This suggests that loss of SCFA in the lower gastrointestinal tract might contribute to pathological manifestations and symptoms in Parkinson’s disease. Our results showed increased α-synuclein expression and release in STC-1 induced by butyrate. Moreover, previous studies showed that administration of a mixture of SCFA (acetate, propionate and butyrate) to germ-free overexpressing α-synuclein mice induced immature microglia and aggregates of α-synuclein in the caudate nucleus, putamen and substantia nigra.^12^ Therefore, the exact role of SCFA in the pathogenesis of Parkinson’s disease, within the gastrointestinal tract and brain, still needs to be elucidated. STC-1 cells are derived from the duodenum, and, to our knowledge, there is no study that has evaluated the composition of the microbiome in the small intestine. Duodenal cells would not normally be exposed to high levels of butyrate since it is produced in the colon of healthy individuals. However, people with Parkinson’s disease suffer from small intestinal bacterial overgrowth^55^ and which bacterial species predominant in the upper gastrointestinal tract of Parkinson’s disease patients and their effect on enteroendocrine cells is unknown.

There are some limitations associated with this work. First, our *in vitro* model evaluated the effect of acute (24 h), high-dose exposure to LPS, PAM and single SCFA (not in combination) rather than a chronic, low-dose exposure. Second, these experiments do not address the pathogenic mechanisms underlying α-synuclein accumulation and/or release (e.g. free or contained in exosomes) or where in the cell it accumulates (cytosolic or vesicular). Moreover, only the monomeric form of α-synuclein was analysed by western blot. However, one would not expect significant aggregation of α-synuclein in 24 h based on the time course of the development of pathology in cultured neurons exposed to, for example, preformed fibrils of α-synuclein,^56^ and we also need to consider that specific, critical concentrations of α-synuclein are needed to promote α-synuclein aggregation.^57^ Another limitation was that we did not evaluate direct markers of TLR activation, such as nuclear translocation of p65 and transcription of NF-kB. Nevertheless, we showed for the first time that LPS, lipopeptides and SCFA trigger α-synuclein pathology in enteroendocrine cells and that specific antagonists to TLR4, TLR2 and FFA receptors prevented the pathological changes. These receptors may therefore become targets of interest for research into future treatments. Our study did not evaluate the potential mechanisms by which α-synuclein pathology can spread from the enteroendocrine cells to the brain. Enteroendocrine cells are polar epithelial cells, and determining whether the release of α-synuclein is global, into the gut lumen from the apical surface of the cell or through synaptic connections onto cells of the enteric nervous system, is important to establish. We believe that our findings can stimulate the design of future studies evaluating enteroendocrine cells as the first site of α-synuclein pathology under specific gut microbial conditions, in particular within the ‘gut-first’ subtype of Parkinson’s disease.

## Supplementary Material

fcad285_Supplementary_Data

## Data Availability

Data and methods that support the findings of this study are openly available at https://zenodo.org and https://www.protocols.io repositories, respectively, as referenced via DOI numbers throughout this manuscript.
