# Supplementary material for: α-Synuclein expression in response to bacterial ligands and metabolites in gut enteroendocrine cells: an in vitro proof of concept study
Source: Brain Commun. 2023 Oct 24;5(6):fcad285. doi: 10.1093/braincomms/fcad285 (PMC10636561; doi:10.1093/braincomms/fcad285)
Supplement: fcad285_Supplementary_Data [file fcad285_supplementary_data.docx]

**Supplementary Figure 1**

**Supplementary Figure 1 legend**

No differences in the number of viable cells were found after 24 hours exposure of STC-1 cells to two concentrations of LPS and PAM (Dunnett’s multiple comparison). Data are presented as box (25^th^ and 75^th^ percentiles) and whisker (5^th^ and 95^th^ percentiles), median (line), mean (+) and all data points.

**Supplementary Figure 2**


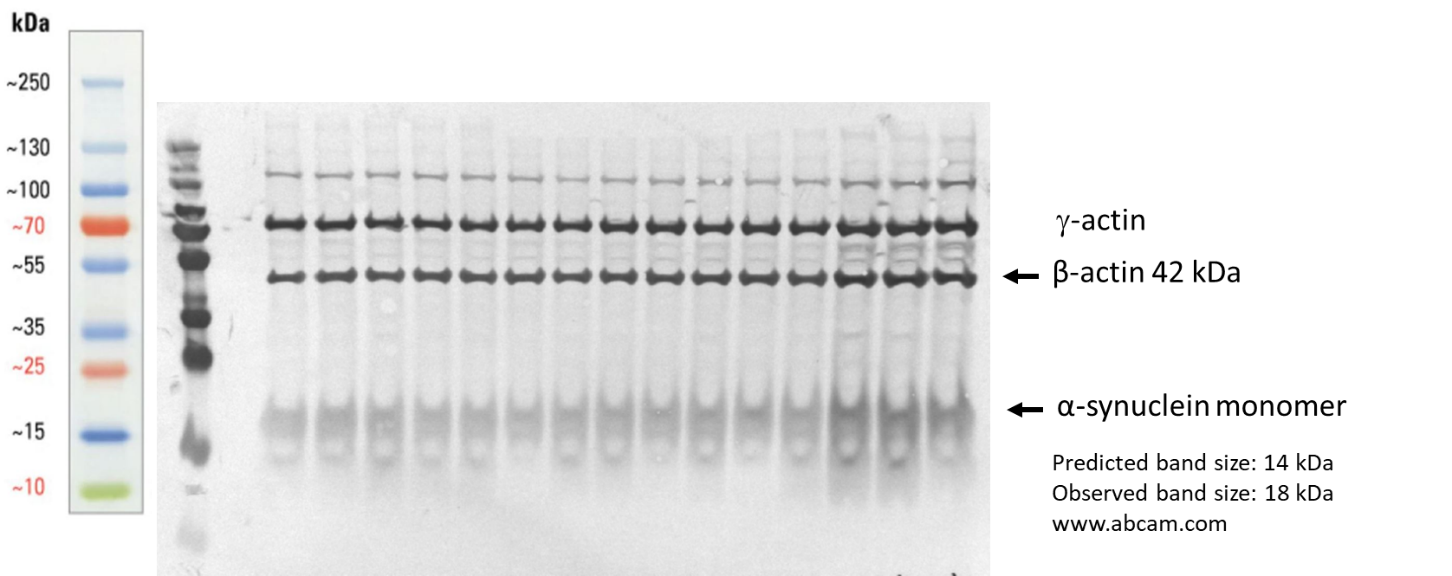


M 1 2 3 4 5 6 7 8 9 10 11 12 13 14 15

**Supplementary Figure 2 legend**

Representative blot used for semi-quantitative measurement of the level of α-synuclein protein monomer in STC-1 cells treated with butyrate and beta-hydroxybutyrate (lanes 1-6), beta-hydroxybutyrate (lanes 7-12) and vehicle (lanes 13-15). M = marker.

**Supplementary figure 3 TLR agonists activate the ubiquitin-proteosome system**


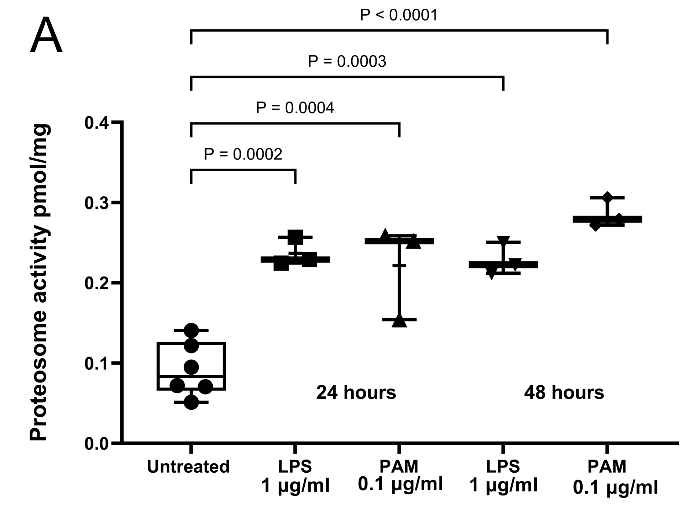


**Supplementary figure 3 legend**

TLR agonists increased proteosome activity (**A**) Stimulation with LPS 1 µg/ml significantly increased proteosome activity after 24 hours (P = 0.0002) and 48 hours (P = 0.0004). Stimulation with 0.1 µg/ml PAM significantly increased proteosome activity after 24 hours (P = 0.0003) and 48 hours P < 0.0001). Data are presented as box (25^th^ and 75^th^ percentiles) and whisker (5^th^ and 95^th^ percentiles), median (line), mean (+) with all data points and were analysed by ANOVA and Dunnett’s multiple comparison test.
